# Respiratory Presentation of Pediatric Patients in the 2014 Enterovirus D68 Outbreak

**DOI:** 10.1155/2016/8302179

**Published:** 2016-08-16

**Authors:** Georgina Martin, Rachel Li, Victoria E. Cook, Matthew Carwana, Peter Tilley, Laura Sauve, Patrick Tang, Akshat Kapur, Connie L. Yang

**Affiliations:** ^1^Department of Pediatrics, University of Saskatchewan, Saskatoon, SK, Canada S7N 0W8; ^2^Department of Pediatrics, British Columbia Children's Hospital, Vancouver, BC, Canada V6H 3V4; ^3^Division of Allergy and Clinical Immunology, British Columbia Children's Hospital, Vancouver, BC, Canada V6H 3V4; ^4^Pathology & Lab Medicine, British Columbia Children's Hospital, Vancouver, BC, Canada V6H 3V4; ^5^Division of Infectious Diseases, British Columbia Children's Hospital, Vancouver, BC, Canada V6H 3V4; ^6^British Columbia Centre for Disease Control, Vancouver, BC, Canada V5Z 4R4; ^7^Division of Respiratory Medicine, British Columbia Children's Hospital, Vancouver, BC, Canada V6H 3V4

## Abstract

*Background*. In the fall of 2014, a North American outbreak of enterovirus D68 resulted in a significant number of pediatric hospital admissions for respiratory illness throughout North America. This study characterized the clinical presentation and risk factors for a severe clinical course in children admitted to British Columbia Children's Hospital during the 2014 outbreak.* Methods*. Retrospective chart review of patients with confirmed EV-D68 infection admitted to BCCH with respiratory symptoms in the fall of 2014. Past medical history, clinical presentation, management, and course in hospital was collected and analyzed using descriptive statistics. Comparison was made between those that did and did not require ICU admission to identify risk factors.* Results*. Thirty-four patients were included (median age 7.5 years). Fifty-three percent of children had a prior history of wheeze, 32% had other preexisting medical comorbidities, and 15% were previously healthy. Ten children (29%) were admitted to the pediatric intensive care unit. The presence of complex medical conditions (excluding wheezing) (*P* = 0.03) and copathogens was associated with PICU admission (*P* = 0.02).* Conclusions*. EV-D68 infection resulted in severe, prolonged presentations of asthma-like illness in the hospitalized pediatric population. Patients with a prior history of wheeze and preexisting medical comorbidities appear to be most severely affected, but the virus can also cause wheezing in previously well children.

## 1. Introduction

Enterovirus D68 (EV-D68) is a nonpolio human enterovirus that shares some biologic features with human rhinoviruses [1, 2]. It was first described in 1962 in association with pediatric respiratory illness [3] but was rarely reported as a cause of human disease until 2008 [4, 5]. Since then, EV-D68 has emerged as a notable pathogen, causing clusters of respiratory illness in Asia, Europe, and the United States [5–8]. The United States National Enterovirus Surveillance System reported 79 cases of EV-D68 from 2009 to 2013, over double the number it reported in the prior three decades [9].

EV-D68 has been associated with a range of respiratory presentations including upper respiratory tract infection, pneumonia, bronchiolitis, bronchitis, and asthma exacerbations [5–8, 10, 11]. The pediatric population appears to be disproportionately affected and the virus can be associated with severe respiratory disease in children [5, 7, 11]. During an outbreak in the United States in 2009, over half of EV-D68 cases detected in children affected those less than four years of age, and 54% resulted in pediatric intensive care unit (PICU) admission [5]. Similarly, a Japanese case series noted that a disproportionate number of pediatric EV-D68 patients presented with an asthma attack that was classified as severe (43.2%) compared to children admitted to the same hospital for asthma exacerbations of other etiologies, in whom only 14.3% were classified as severe [11].

In the fall of 2014, an outbreak of EV-D68 resulted in numerous pediatric hospital admissions for respiratory illness throughout Canada and the United States, with documented cases in 49 American states and nine Canadian provinces [12]. Surveillance data revealed that affected individuals presented with symptoms of lower respiratory tract illness and that children with preexisting asthma were particularly at risk [9, 12, 13]. Despite its increasing prevalence over the past decade, the characteristics of this virus, including the epidemiology and spectrum of illness, are not yet well described [14]. The current study seeks to characterize cases of EV-D68 admitted to British Columbia Children's Hospital (BCCH) from September to December of 2014. We describe the patient demographics, clinical presentation, management at our institution, and underlying characteristics that may predispose patients to more severe clinical presentations.

## 2. Methods

This retrospective case series included patients aged 0–18 years who were admitted to BCCH with a respiratory illness and tested positive for EV-D68 on a nasopharyngeal sample between August 28 and December 31, 2014. The start date represents the first confirmed case of EV-D68 at BCCH and the end date correlates with the end of the British Columbia Centre for Disease Control's (BCCDC's) enhanced surveillance period for EV-D68. Patients who had nasopharyngeal samples tested for respiratory viruses were identified from the hospital microbiology electronic database. Patients older than 18 years of age, who were not admitted to BC Children's Hospital and who had nonrespiratory presentations, were excluded from the study. The British Columbia Children & Women's Research Ethics Board approved this study.

Nasopharyngeal washings (NPW) or nasopharyngeal FLOQ swabs® (Copan, Murrieta CA USA) were tested by VIRAP (panel of direct fluorescent antibody stains covering RSV, influenza A, influenza B, parainfluenza 1, parainfluenza 2, parainfluenza 3, adenovirus, and human metapneumovirus). Samples were tested further by polymerase chain reaction (PCR) upon physician request, for a panel of respiratory pathogens (RSV, influenza A and influenza B, parainfluenza 1, parainfluenza 2, parainfluenza 3, adenovirus, human metapneumovirus, coronaviruses, enterovirus, rhinovirus,* Mycoplasma pneumonia*,* Chlamydophila pneumonia*,* Streptococcus pneumonia*, and* Bordetella pertussis*) or after October 4 a single PCR for enterovirus could also be requested (TrimGen Enterovirus Detection Kit® (TrimGen, Sparks, Maryland) or an in-house TaqMan respiratory PCR panel containing singleplex primers and probes for enteroviruses, rhinoviruses, and enterovirus species D). Testing was ordered at the discretion of the treating physician, typically for children admitted with respiratory symptoms of work of breathing or wheeze, and a specific case definition was not used. All specimens positive for an enterovirus were forwarded to the British Columbia Public Health Microbiology and Reference Laboratory (BC PHMRL) for typing. A second EV-D68-specific qRT-PCR was performed at the BC PHMRL to identify all cases of EV-D68.

A standardized data collection tool was used to collect data from patient charts including demographic information, medical history, clinical presentation, investigations, and hospital course. Chest radiographs were reviewed by a pediatric respirologist (CY) and findings were classified by appearance as minor patchy changes, major patchy changes, lobar changes, peribronchial thickening, and hyperinflation. Data was entered into a database and descriptive statistics were used to characterize findings. Where appropriate, variables were compared using chi-square, two-tailed *t*-tests and Wilcoxon rank sum tests to determine statistical significance.

## 3. Results

**Figure 1:**
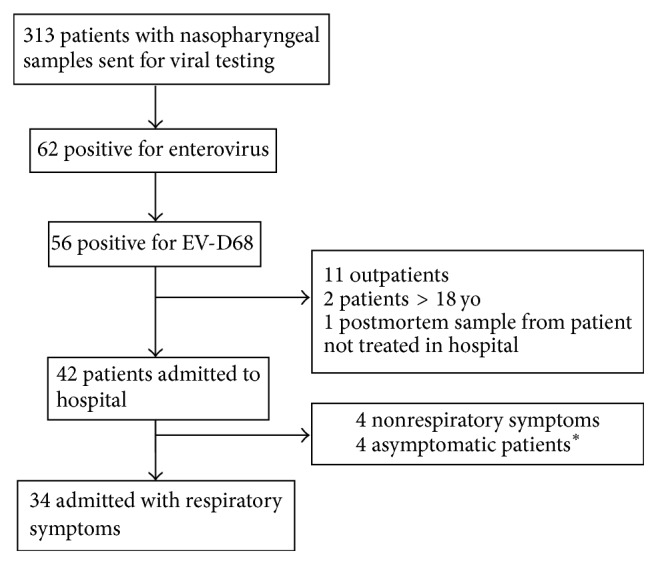
Flowchart of participants. ^*∗*^3 patients had swabs as part of a presurgical assessment and 1 patient was to start chemotherapy and had a swab because of remote viral symptoms.

**Figure 2:**
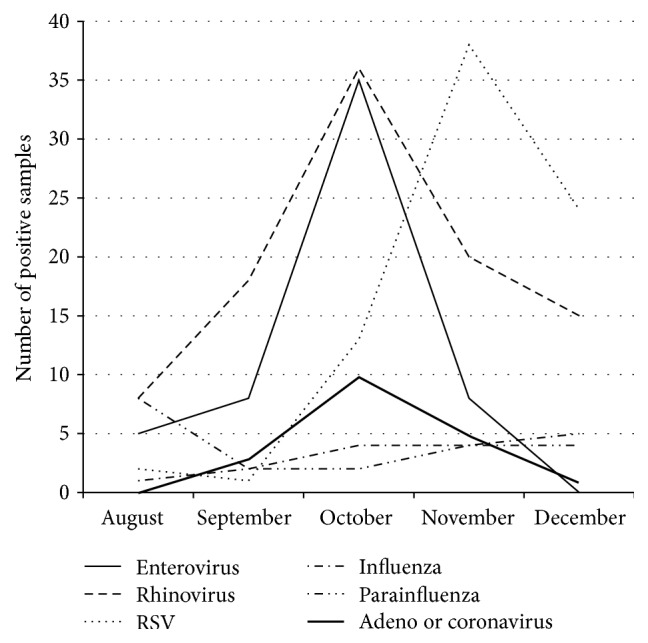
Respiratory viruses isolated from nasopharyngeal samples, 2014.

A total of 876 nasopharyngeal samples were collected and tested for viruses at the BCCH laboratory during the study period. Of these, 62 were positive for enterovirus and 56 (6.4%) were subtyped as EV-D68 at the reference laboratory (BCP HMRL) (Figure 1). Other common viruses during the study period included rhinovirus, influenza, and towards the end of the study period respiratory syncytial virus (RSV) (Figure 2).

Eleven cases were excluded because they were outpatients, and two cases were excluded because they did not fulfill age criteria. One case was a sample sent from the morgue and was excluded because the child was never admitted at BCCH. Of the remaining 42 patients who were admitted to our institution, eight were excluded because their presentation was nonrespiratory. Those with a nonrespiratory presentation included three children who presented with acute flaccid paralysis, one with a gastrointestinal bleed, three asymptomatic patients who were positive on presurgical screening, and one oncology patient who had a remote history of viral symptoms. In total, 34 patients met criteria and were included in the study.

### 3.1. Patient Characteristics and Clinical Presentation

Children were distributed across all age groups, with the majority being over the age of 5 years (60%) (Table 1). Fifty-three percent had a past medical history of parent reported wheeze but were otherwise healthy. A further third of children (32%) had other medical comorbidities including congenital heart disease, aspiration lung disease, cerebral palsy, prematurity, and chromosomal abnormalities. Two children were on home oxygen (one with trisomy 21, aspiration lung disease, and an AVSD and the other with aspiration lung disease and tetralogy of Fallot), and one child had a tracheostomy and was on a home ventilator (for central hypoventilation).

Two-thirds (65%) of children had had prior presentations to the hospital with respiratory distress or wheezing, and 35% had been previously admitted to the hospital for respiratory illness. The five previously healthy children, who had no history of wheezing, had a median age of 7.5 years (range 3–10 years) and presented with shortness of breath and wheezing.

Most children presented in the month of October. Typically, children presented with a prodrome of three days, though duration of symptoms ranged from one to seven days (Table 2). The most common symptoms reported by families were shortness of breath (82%), cough (82%), and rhinorrhea or nasal congestion (71%). On physical exam, children were tachypneic (79%) or tachycardic (59%) or had oxygen saturations less than 92% (59%). Common auscultatory findings included decreased air entry (88%) and wheeze (76%). A Paediatric Respiratory Assessment Measure (PRAM) score was done on 22 patients and all were classified as having severe (73%) or moderate (27%) distress [16].

Twenty-six children had a full respiratory pathogen PCR panel done, while the remaining eight children had testing done for enteroviruses but were not tested for other viruses. Copathogens were identified in 42% of children who had a full respiratory pathogen panel performed, with* Streptococcus pneumoniae* [5], rhinovirus [2], adenovirus [2], and influenza [2] being the most commonly identified organisms. The most common abnormal findings on chest X-ray were peribronchial thickening (38%) and hyperinflation (31%). Forty-five percent had evidence of airspace disease, primarily in the form of minor (21%) and major (21%) patchy changes.

### 3.2. Management and Clinical Course

In the emergency room, 65% of children were started on our institution's asthma protocol, which involved PRAM scoring, oral dexamethasone, and three rounds of salbutamol and ipratropium spaced 20 minutes apart. An additional 18% of children received salbutamol alone. Of the 22 children who were treated on the asthma protocol, 20 had repeat PRAM scoring after bronchodilators (Table 3). Forty-five percent of these children improved (defined as a reduction in the PRAM score of ≥3) after initial treatment [16]. The remaining 55% did not improve with bronchodilators. Response to bronchodilators did not vary with age (*P* = 0.14) or gender (*P* = 0.58) and did not correlate with parent reported history of wheeze (*P* = 0.80).

Ten patients (29%) were admitted to the pediatric intensive care unit for respiratory support: seven for high flow oxygen, two for bilevel positive airway pressure (BiPAP), and one for adjustment of tracheostomy settings. Children admitted to the ICU were more likely to have a medical comorbidity (60% versus 21%, *P* = 0.03) and to have at least one copathogen on PCR testing of the nasopharyngeal sample (70% versus 17%, *P* = 0.02). Otherwise, they did not differ significantly from those admitted to the ward (Table 4).

Median length of hospital stay for all patients was 90 hours (range 22 to 432 hours). Patients with a history of wheezing and other medical comorbidities and who were previously healthy had a median length of stay of 82.5 (range 31–148), 161 (range 37–432), and 50 (range 22–96) hours, respectively (*P* = 0.016).

## 4. Discussion

During the fall of 2014, there were 34 pediatric patients with EV-D68 positive nasopharyngeal samples who were admitted to BCCH for respiratory illness. In our series, the median age was 7.5 years and the most common age group was 5–9-year-olds. Reports from previous EV-D68 outbreaks found that children 0–4 years of age were the most likely to be hospitalized [5, 7]. However, during the 2014 outbreak, it appears that school aged children were disproportionately affected, which is consistent with findings from other studies [13, 17]. In addition, studies from the 2014 outbreak that have compared patients with EV-D68 to non-EV-D68 patients found that cases with EV-D68 were older [13, 17]. Of note, there were only two cases of EV-D68 in infants less than one year at our institution, and both were excluded from this study as they had primarily nonrespiratory presentations. Given that 37% of nasopharyngeal testing in our study was done in the age group of 0-1 year, this finding points to a lower rate of exposure or less severe clinical presentation in infants.

Fifty-three percent of cases in our series had a prior history of parent reported wheeze. This is in keeping with other studies from the 2014 outbreak, which report that 31–70% of children admitted to hospital with EV-D68 had preexisting asthma [9, 11, 13, 17–19]. Our findings contribute to the mounting body of evidence that EV-D68 disproportionately affects those with underlying respiratory disease. However, like the Kansas City cohort, our study also identified 15% of children who were previously healthy, indicating that the virus can also cause significant respiratory disease with wheezing in those with no identifiable risk factors, although none of these children required PICU admission [17].

In our study, one-third of patients had medical comorbidities other than wheeze and comprised 60% of those admitted to the PICU, suggesting a more severe clinical course in this population. Prior reports have found that medically complex and immunosuppressed adult patients may be more susceptible to EV-D68 [6, 19, 20], and there has been at least one report of a death in a medically complex child in the context of EV-D68 infection [21]. A case series of inpatients and outpatients reported a similar rate of 28% with medical comorbidities [18], although another study involving only patients admitted to the PICU found that only 18% had medical comorbidities.

In our series, 30% of patients required admission to the PICU. PICU admission rates reported during the 2014 outbreak were 4% in Alberta, 18% in Kansas City, and 23% in Hamilton [13, 17, 18]. Variation in reported rates may be explained by differences in patient populations, as the Hamilton and Alberta studies included both inpatients and outpatients. Of note, all children admitted to the PICU in our study received high flow oxygen, CPAP, or BiPAP; in contrast, only 51% and 10% of PICU admitted children in Kansas City and Hamilton received either noninvasive or invasive ventilation [17, 18]. Thus, indications for PICU admission appear to vary across sites and may be another reason for the discrepancy in rates.

When a full respiratory pathogen PCR panel was done, 42% of patients had copathogens identified on testing of their nasopharyngeal sample. A high rate of copathogen presence has also been observed in prior EV-D68 outbreaks [22, 23]. Whilst potentially pathogenic bacteria can colonize the nasopharynx without causing disease, there is some evidence that bacterial colonization may have a role in recurrent wheezing episodes either independently or as a cofactor to viral infection [24, 25]. A recent meta-analysis did not reveal any difference in clinical disease severity between children with viral coinfections and those with a single pathogen [26]. However, in our case series, children admitted to the PICU were significantly more likely to have an identified respiratory copathogen suggesting that the role of copathogens in EV-D68 infection merits further inquiry.

Our study was limited by the fact that it was retrospective and thus, the decision to do viral testing was at the clinician's discretion. We did not have a control group and cannot comment on the severity of infection compared to children with other respiratory viruses. Furthermore, our sample size was small and excluded those who were managed as outpatients, likely overestimating the severity of EV-D68 infection.

This study adds to the growing body of evidence that EV-D68 causes significant respiratory disease in children, even in those with no previous respiratory illness. Further studies are needed to delineate the long term sequelae of the virus in this group. In addition to those with a previous history of wheezing, this study also found that those with complex medical conditions are at risk for severe illness related to EV-D68. Finally, ongoing epidemiologic surveillance is needed to monitor the evolution of the virus as a significant human pathogen.

## Figures and Tables

**Table 1:** Characteristics of hospitalized children testing positive for EV-D68 (*n* = 34).

Patient characteristics	*n* (%)
*Age*	
<2	5 (15)
2–4	7 (21)
5–9	13 (38)
10–14	5 (15)
>15	4 (12)

*Gender*	
Male	19 (56)

*Past medical history*	
History of parent reported wheeze, but otherwise healthy	18 (53)
Medical comorbidities aside from wheeze^*∗*^	11 (32)
Healthy (no wheeze or medical comorbidities)	5 (15)

*Prior presentations to hospital for respiratory distress*	
None	12 (35)
Emergency room only	10 (30)
Admitted to hospital	12 (35)
Admitted to ICU	5 (15)

^*∗*^Medical comorbidities include children with congenital heart disease, chromosomal abnormalities, aspiration lung disease, dysautonomia, prematurity, and cerebral palsy.

**Table 2:** Clinical presentation of hospitalized children with EV-D68.

Clinical presentation	*n* (%)
*Month of presentation (total n* = 34)	
August	1 (3)
September	5 (15)
October	25 (74)
November	3 (9)

*Parent reported symptoms at presentation (total n* = 34)	
Shortness of breath	28 (82)
Cough	28 (82)
Rhinorrhea/congestion	24 (71)
Fever	21 (62)
Wheeze	15 (44)
Vomiting	11 (32)

*Vital signs at presentation (total n* = 34)	
Tachypnea^*∗*^	27 (79)
Tachycardia^*∗*^	20 (59)
Oxygen saturation <92%	20 (59)
Fever ≥ 38°C	4 (12)

*Auscultatory findings (total n* = 34)	
Decreased air entry	30 (88)
Wheeze	26 (76)
Crackles	12 (35)
Silent chest	9 (26)

*PRAM score at presentation (total n* = 22)	
0–3 (mild)	0 (0)
4–7 (moderate)	6 (27)
8–12 (severe)	16 (73)

*Laboratory findings (total n* = 23)	
Leukocytosis^*∗∗*^	12 (52)
Neutrophilia^*∗∗*^	17 (74)
Lymphopenia^*∗∗*^	19 (83)

*Copathogens identified on PCR testing of nasopharyngeal washings (total n* = 26)	11 (42)

*Chest X-ray findings (total n* = 29)	
Normal chest X-ray	4 (14)
Minor patchy changes	6 (21)
Major patchy changes	6 (21)
Lobar changes	1 (3)
Peribronchial thickening	11 (38)
Hyperinflation	9 (31)

^*∗*^Vital sign parameters were defined according to norms for age as per the Hospital of Sick Children's Handbook of Pediatrics [15].

^*∗∗*^As defined by BCCH's laboratory reference values for age.

**Table 3:** Hospital course of pediatric inpatients with EV-D68.

Hospital course	*n* (%)
*Location of initial admission *	
Pediatric intensive care unit	10 (29)
Inpatient ward	24 (71)

*Maximal respiratory support*	
No oxygen	11 (32)
Low flow oxygen	13 (38)
High flow oxygen	7 (21)
BiPAP	2 (6)
Adjusted home tracheostomy settings	1 (3)

*Therapeutic modalities *	
Systemic corticosteroids	30 (88)
Systemic antimicrobials	16 (47)
Magnesium sulfate bolus	13 (38)
Aminophylline infusion	4 (12)

*Corticosteroid use*	
Methylprednisolone IV	14 (47)
Dexamethasone or prednisolone PO	16 (53)

**Table 4:** Characteristics of children admitted to the PICU compared to children who did not require PICU admission.

	Admitted to ICU	No ICU admission	*P* value
	*n* (%)	*n* (%)	
	Total *n* = 10 unless otherwise noted	Total *n* = 24 unless otherwise noted	
Mean age	7.4 years	7.1 years	*P* = 0.89

Male gender	5 (50)	14 (58)	*P* = 0.66

Initial PRAM (moderate/severe)	0, moderate	6, moderate (32)	*P* = 0.25
	3, severe (100)	13, severe (68)	
	(total *n* = 3)	(total *n* = 19)	

Lymphopenia	7 (78)	12 (50)	*P* = 0.90
	(total *n* = 9)	(total *n* = 15)	

History of wheezing	8 (80)	16 (67)	*P* = 0.44

Other medical comorbidities^*∗*^	6 (60)	5 (21)	*P* = 0.03

Copathogen present on full respiratory virus PCR panel^*∗∗*^	7 (70)	4 (25)	*P* = 0.02
	(total *n* = 10)	(total *n* = 16)	

^*∗*^Medical comorbidities include children with congenital heart disease, chromosomal abnormalities, aspiration lung disease, dysautonomia, prematurity, and cerebral palsy.

^*∗∗*^Copathogens in ICU patients: adenovirus, *Streptococcus pneumonia*, and influenza A; copathogens in non-ICU patients: *Streptococcus pneumonia*, influenza B, and rhinovirus.
